# Supplementary material for: Comparison of Methods for Incorporating Related Data When Developing Clinical Prediction Models: A Simulation Study
Source: Biom J. 2026 Aug 27;68(5):e70166. doi: 10.1002/bimj.70166 (PMC13520826; doi:10.1002/bimj.70166)
Supplement: Supplementary file 2 [file BIMJ-68-e70166-s001.zip › third submission code review - R2/reproducibility_supplement/results/Plots/validation_mterics_plot_combination_SC_ID8_TS0.25_DS4_model15.pdf]

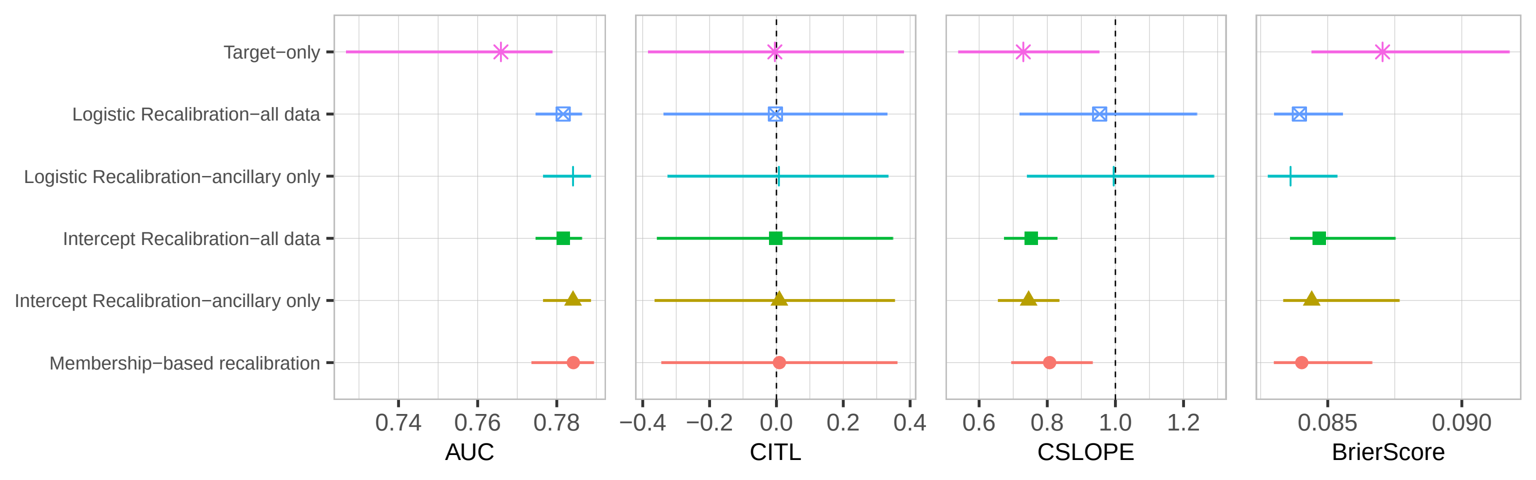
Target−only
Logistic Recalibration−all data
Logistic Recalibration−ancillary only
Intercept Recalibration−all data
Intercept Recalibration−ancillary only
Membership−based recalibration
0.74
0.76
0.78
AUC
−0.4
−0.2
0.0
0.2
0.4
CITL
0.6
0.8
1.0
1.2
CSLOPE
0.085
0.090
BrierScore
